# Unveiling the role of aldosterone in metabolic dysfunction–associated steatotic liver disease

**DOI:** 10.1007/s11154-026-10056-3

**Published:** 2026-06-30

**Authors:** Anna Hernández-Rubio, Maria-Teresa Julián, Elena Valassi, Nuria Alonso, Manel Puig-Domingo

**Affiliations:** 1 Department of Medicine, Autonomous University of Barcelona, Carretera de Canyet s/n, 08916 Badalona, Spain; 2 Department of Endocrinology and Nutrition, Germans Trias i Pujol University Hospital, Badalona, Spain; 3 Germans Trias i Pujol Research Institute, Barcelona, Spain

**Keywords:** Aldosterone, Metabolic dysfunction-associated steatotic liver disease, Liver fibrosis, Metabolic syndrome, Renin-angiontensin system

## Abstract

Metabolic dysfunction-associated steatotic liver disease (MASLD) is an increasing global health challenge closely linked to obesity and the metabolic syndrome. This review explores the role of aldosterone as a central contributor to metabolic dysfunction, highlighting its association with adipose tissue dysfunction and systemic insulin resistance which can further influence MASLD development and progression. While aldosterone-related organ damage has been extensively studied in other contexts such as kidney disease, its involvement in MASLD has received less attention. Beyond classical renin-angiotensin-aldosterone system activation, aldosterone secretion can be directly stimulated by leptin and other adipogenic factors, creating a pathogenic link between obesity and metabolic impairment. Aldosterone amplifies chronic inflammation, oxidative stress and insulin resistance, primarily through mineralocorticoid receptor (MR)-dependent mechanisms. These involve both genomic actions regulating pro-inflammatory genes and rapid non-genomic signalling. Additionally, aldosterone MR-independent effects, can further contribute to oxidative stress and inflammatory responses. Significantly, the MR is expressed in liver cells, including hepatocytes, liver sinusoidal endothelial cells, Kupffer cells, and hepatic stellate cells. This expression can allow for direct aldosterone-mediated effect on hepatic inflammation, oxidative stress, and fibrosis, which can occur alongside or independently of systemic metabolic pathways. Such direct mechanisms may explain associations found in clinical studies correlating high aldosterone levels -even with low renin- with the prevalence and progression of MASLD. Recognizing aldosterone as a pleiotropic hormone that integrates metabolic and hepatic disease offers new insights into the cardio-kidney-metabolic syndrome and underscores the potential for targeted therapies.

## Introduction

Metabolic dysfunction-associated steatotic liver disease (MASLD) is currently the most common chronic liver disease globally, affecting over one third of the adult population. MASLD prevalence and burden of disease is increasing along with the increase in the prevalence of obesity, type 2 diabetes and metabolic syndrome [1].

Hepatic consequences of MASLD are associated with its progress to steatohepatitis, fibrosis, advanced fibrosis, cirrhosis and/or liver cancer. However, MASLD is also closely associated with extrahepatic complications, where cardiovascular events are the leading cause of death in this population [2].

Adipose tissue dysfunction and insulin resistance are key drivers in MASLD, which can lead to increased flux of free fatty acids to the liver, accumulation of toxic lipid species and lipotoxicity. Lipotoxicity induces endoplasmic reticulum stress, mitochondrial dysfunction and oxidative stress, ultimately triggering hepatocyte apoptosis and inflammation [3]. Genes involved in lipid metabolism (e.g. variants in PNPLA3, TM6SF2) can modulate susceptibility and disease severity, interacting with environmental factors such as diet, endocrine chemical disruptors and physical activity [4]. These processes form the basis of metabolic syndrome, which includes glycemic alterations, low HDL-cholesterol, hypertriglyceridemia, obesity and/or hypertension; and for MASLD diagnosis at least one of these criteria has to accompany the evidence of liver steatosis [4].

Among the pathogenic mechanisms related to MASLD, the activation of the renin-angiotensin-aldosterone system (RAAS) has been hypothesised to have a role either as a driver and consequence of MASLD, with evidence mainly supported through animal models (mice) and human clinical data predominantly observational [5]. The RAAS cascade starts in the liver, where angiotensinogen is produced, which has been found to be upregulated in cirrhosis and associated with the activation of hepatic stellate cells promoting fibrogenesis [6]. In addition, adipocytes can also secrete adipokines and angiotensinogen, both of which contribute to sympathetic nervous system and RAAS activation [7, 8]. Angiotensinogen regulation varies across sites, where different gene transcription factors are present in liver and adipocytes [9]. Renin cleaves angiotensinogen to form angiotensin I, and is released in response to decreased renal perfusion, reduced sodium delivery, or sympathetic activation. Importantly, renin operates below substrate saturation at physiological levels, making angiotensinogen a key limiting driver of RAAS activity. Angiotensin I is then converted by angiotensin converting enzyme (ACE) into angiotensin II, which stimulates aldosterone synthesis in the adrenal cortex [9, 10]. These mechanisms create a synergistic-amplifying loop where metabolic dysfunction and lipid accumulation activate intrahepatic RAAS, angiotensin II promotes further lipid accumulation, oxidative stress and inflammation, worsening steatohepatitis and fibrosis, ultimately perpetuating metabolic dysfunction and RAAS activation [11, 12]. Additionally, these alterations can also lead to the development of endothelial dysfunction, vascular stiffness and hypertension, especially through aldosterone, the final metabolite of the cascade [12–14].

Aldosterone is a mineralocorticoid hormone produced in the zona glomerulosa of the adrenal cortex from cholesterol -which is converted to intermediary metabolites and finally aldosterone through the aldosterone synthase enzyme (CYP11B2)-. The main function of aldosterone is to regulate salt, water balance, and blood pressure. Its secretion is stimulated through the RAAS activation -as already mentioned-, elevated serum potassium and adrenocorticotropic hormone [15]. Aldosterone acts mainly via the mineralocorticoid receptor (MR) which is the primary receptor for aldosterone effects, although MR-independent pathways have also been described. The MR, encoded by the NR3C2 gene, is a member of the nuclear receptor superfamily of ligand-dependent transcription factors. Its activation is not exclusively dependent on aldosterone, as cortisol binds the MR with comparable affinity. The enzyme 11β-hydroxysteroid dehydrogenase (HSD) regulates the activation of cortisol, as 11β-HSD1 catalyses the conversion of inactive cortisone to active cortisol, and 11β-HSD2 mediates the reverse reaction, thereby limiting cortisol availability. Tissues with low 11β-HSD2 expression, such as the liver, increased cortisol production mediated by 11β-HSD1 can be present so that MR activation could be substantially influenced by cortisol rather than aldosterone. This tissue-specific balance has important implications for interpreting the complexity of MR activation, as endogenous cortisol may confound or modulate the apparent contribution of aldosterone to MR-dependent pathways [16].

When aldosterone binds the MR it can act through a classical genomic pathway translocating to the nucleus where regulates the expression of multiple genes involved in ion transport in the renal tubular epithelium, including the epithelial sodium channel (ENaC) and the Na⁺/K⁺-ATPase, thereby promoting sodium and water reabsorption and potassium excretion. This pathway predominates under physiological conditions providing sustained responses for maintaining sodium/volume homeostasis. Simultaneously, when aldosterone binds MR, there are other rapid non-genomic effects that are produced to give immediate response to blood pressure regulation (e.g., rapid vasoconstriction and phosphorylation of existing ENaC, among others). Finally, a MR-independent non-genomic pathway is relevant specially in pathological conditions which include aldosterone excess and will be further discussed.

Elevated aldosterone may occur in the setting of high renin -secondary aldosteronism- or low renin -primary aldosteronism (PA)-, the latter typically due to aldosterone-producing adenomas or hyperplasia and sometimes accompanied by cortisol co-secretion which can enhance organ damage in PA. Clinical consequences of PA include hypertension (often drug-resistant), decreased plasma renin activity and hypokalemia. Regardless of blood pressure, PA has been associated with increased cardiovascular and renal morbidity [17]. Management of PA involves treatment with mineralocorticoid receptor antagonists (e.g., spironolactone, eplerenone), and sometimes adrenalectomy when unilateral disease is confirmed. Additionally, novel aldosterone synthase inhibitors have shown promising results in PA management in phase 3 clinical trials [18].

From a clinical perspective, aldosterone excess beyond PA drives myocardial remodelling, vascular fibrosis, endothelial dysfunction, and inflammation [19]. These alterations may result in myocardial fibrosis with impaired diastolic function, vascular stiffening, increased stroke risk, and renal damage marked by hyperfiltration and glomerulosclerosis leading to chronic kidney disease. MASLD and renal disease are hallmark manifestations of the cardio-kidney-metabolic syndrome, arising from adipose tissue dysfunction and a cascade of insulin resistance, chronic inflammation, and neurohormonal activation [20]. Within this interconnected metabolic framework, aldosterone excess has emerged as a potential contributor to metabolic dysregulation.

The association between aldosterone excess and metabolic syndrome has been studied previously having positive associations in clinical cross-sectional studies [21, 22]. However, whether the associations are causal, or mediated through other confounding factors (e.g.: renin-dependent aldosteronism, co-secretion of cortisol) remains to be elucidated [22].

Finally, the association between aldosterone and liver disease, and more specifically with MASLD has been less investigated. Preclinical and translational studies indicate that pharmacological inhibition of RAAS may attenuate liver fibrosis and inflammation through its effects on hepatic stellate cells and vascular function [23]. Hepatic stellate cells are key mediators of fibrosis through its activation into collagen-producing myofibroblasts and evidence has shown in animal models that RAAS and angiotensin-II inhibitors reduce its activation supressing pro-fibrotic signalling pathways [24–26]. Further research is needed to confirm these findings in clinical populations with MASLD.

The present review aims to provide a comprehensive updated overview on the potential influence of aldosterone in MASLD, by analysing current evidence of its association with each metabolic syndrome component and MASLD. Ultimately, we aim to provide potential mechanistic insights of the evidence on sex differences. We believe that this review will help detect gaps of knowledge and pave the way for identifying novel pathogenic mechanisms and potential treatment targets in MASLD.

## Aldosterone and metabolic syndrome components

Aldosterone is a pleiotropic hormone with a systemic role beyond blood pressure regulation. The MRs are present in several tissues such as endothelial cells, adipocytes, immune cells, muscle, pancreatic β-cells, and hepatic cells, among others. The activation of MRs through aldosterone binding in different tissues has been associated with endothelial dysfunction, oxidative stress, inflammation and fibrosis, leading to vascular ageing and organ damage.

Aldosterone binds to the cytoplasmic MR where it has a genomic action by translocating to the nucleus and regulating gene transcription. In the nucleus, aldosterone-MR complex acts as a ligand-activated transcription factor, binding to specific DNA sequences known as MR response elements in the promoter regions of target genes. Classical genes expressed include ENaC-Na/K ATPase which regulates sodium and water balance, but other pro-inflammatory and pro-fibrotic genes can be activated and contribute to inflammation, vascular and metabolic dysfunction. These include Plasminogen Activator Inhibitor-1 (PAI-1), Transforming Growth Factor-β (TGF-β), Endothelin-1 (ET-1) and Connective Tissue Growth Factor (CTGF), among others [27]. On the other hand, aldosterone can act through non-genomic pathways with rapid cellular effects not requiring gene transcription or protein synthesis. These effects can be mediated by MR-dependent (mainly) and MR-independent mechanisms. MR-dependent non-genomic mechanisms include the activation of kinase signalling cascades, increased intracellular calcium and phosphorylation of MR itself [19]. MR-independent pathways involve other molecules such as the G-protein coupled estrogen receptor (GPER), Caveolin-1 and striatin scaffolding proteins, which can lead to reactive oxygen species production and inflammation [28]. However, it should be taken into account that there’s a substantial cross-talk between all these mechanistic pathways, and all of them are relevant in contributing to cardio-kidney-metabolic diseases [19].

Additionally, beyond RAAS activation, aldosterone secretion and consequent MR activation can be enhanced by mediators produced by other tissues, such as leptin derived from adipose tissue, as discussed below [29, 30]. All this together, can contribute to insulin resistance, dyslipidemia and adipose tissue dysfunction - core components of the metabolic syndrome. In parallel, aldosterone-mediated endothelial dysfunction and activation of the RAAS accelerates cardiovascular remodelling and impair renal function, thereby linking metabolic dysregulation with the cardio-kidney metabolic syndrome, where elevated aldosterone levels and MR overactivation are increasingly recognized unifying mechanisms [19].

Below we discuss evidence on the association between aldosterone excess and each component of the metabolic syndrome.

### Aldosterone and adipose tissue dysfunction

The link between dysfunctional adipose tissue and activation of the RAAS was described more than 10 years ago as evidenced in the studies referenced throughout this manuscript [31]. Adipocytes have a well-known classic endocrine role through the secretion of adipokines including leptin which regulates appetite and energy expenditure, and adiponectin which enhances insulin sensitivity and exerts anti-inflammatory effects, but also angiotensinogen can be partially secreted and modulated in human adipose tissue. Evidence in human and mouse adipocytes showed the presence of aldosterone synthase and the ability to secrete aldosterone, which regulates adipocyte differentiation and vascular function in an autocrine and paracrine manner, respectively [32]. There’s also evidence of stimulation of aldosterone independent of RAAS activation, by adipogenic factors, specially leptin [29, 33, 34]. Leptin has been shown to induce aldosterone synthase expression, resulting in aldosterone excess, which has been proposed as a mechanistic link between obesity and hypertension [33, 35]. Non-esterified fatty acids (palmitic acid and linoleic acid), which are in excess in obesity and metabolic syndrome, have also demonstrated to induce the expression of aldosterone synthase subsequently increasing aldosterone levels [36].

On the other hand, weight loss in the context of obesity has been found to be associated with a decrease in plasma aldosterone levels, which reinforces the potential role of aldosterone in adiposity [37–40].

Obesity phenotyping has evolved in the last decade beyond the use of body mass index (BMI) for assessing the cardiometabolic risk, which does not reflect adipose tissue distribution. Adipose tissue can be divided into subcutaneous adipose tissue (SAT) and visceral adipose tissue (VAT), the later playing the main pathogenic role in metabolic diseases. VAT can be further classified between periorgan fat (e.g., perirenal, epicardial) which exerts harmful effects via mechanical compression and local pro-inflammatory signalling, and intraorgan fat (e.g., hepatic steatosis, intramiocardial fat) which mainly drivers lipotoxicity and cellular dysfunction. In clinical practice, VAT is commonly estimated using waist circumference.

Clinical studies on the association between PA and VAT have reported that increased abdominal visceral-to-subcutaneous fat ratio interacts with increased plasma aldosterone con cardiac function [41]. Regarding specific VAT depots, main evidence has been published around perirenal adipose tissue. A recent study from Shen H et al. with prospective data from the UK Biobank, demonstrated an association between increased perirenal adipose tissue, idiopathic hyperaldosteronism and low-renin hypertension, where suggested mechanisms explaining the association included the compression of renal parenchyma and vasculature, as well as paracrine effects through adipokine secretion [42]. Consistently, a study in individuals with PA reported that perirenal adipose tissue accumulation was associated with higher aldosterone levels as well as renal and metabolic disturbances (increased fasting glucose, uric acid, C reactive protein, triglycerides, among others) [43]. In the experimental part of this study, mice treated with MR antagonists exhibited reduced markers of macrophage infiltration and fibrosis in perirenal adipose tissue, suggesting that perirenal fat accumulation may contribute to the mechanisms linking MR activation to cardiometabolic and renal dysfunction in PA [43].

Beyond perirenal fat infiltration, also periadrenal adipose tissue in aldosterone-producing adenomas have been shown to exert endocrine and paracrine effects that may influence the pathophysiology and outcomes of PA [44]. However, data on the association between aldosterone excess and adipose tissue depots at other specific anatomical locations remains limited. Excess aldosterone has been described to disrupt autophagy in perivascular adipose tissue, ultimately driving to inflammation, oxidative stress, vascular impairment and metabolic dysfunction [45, 46].

Taken together, these findings suggest that adipose tissue is involved in aldosterone production and modulation through mechanisms that extend beyond classical RAAS activation.

### Aldosterone, insulin resistance and diabetes

Elevated aldosterone levels, due to PA or activation of the RAAS, have been found associated with development of type 2 diabetes through oxidative stress, inflammation and dysregulation of adipokines, contributing to insulin resistance [47].

Aldosterone excess, acting via the MR, activates kinases such as Src which phosphorylate insulin receptor substrate-1 (IRS-1) at specific serine residues. This aberrant phosphorylation marks IRS-1 for ubiquitination and proteasomal degradation, reducing IRS-1 protein levels in different cells such as adipocytes, vascular smooth muscle, skeletal muscle and myocardiocytes [48]. The loss of IRS-1 disrupts downstream insulin signalling, decreasing Akt phosphorylation and glucose uptake, leading to insulin resistance. This process can be further amplified by aldosterone-induced ROS production [49].

Additionally, vesicle trafficking and membrane fusion of glucose transporter 4 (GLUT4) are disrupted. MR-aldosterone overactivation also decreases GLUT4 gene expression at the nuclear level. Therefore, a reduction of GLUT4 availability at the membrane is observed, decreasing glucose uptake and contributing to systemic insulin resistance [48, 50]. Another mechanism of hyperglycemia has been described through the action of aldosterone at the glucocorticoid receptor level, by increasing the expression of hepatic glucose-6-phosphate dehydrogenase (G6PD), promoting gluconeogenesis and elevating blood glucose levels in mice model receiving aldosterone [51]. This evidence on metabolic consequences mediated by aldosterone through other receptors is especially relevant since this effect can escape from treatment with MR blockers. Finally, the proinflammatory and profibrotic effects of aldosterone can also act in the pancreas, directly producing β-cell dysfunction with decreased insulin production [52, 53]. This dysfunction can be mediated through MR-dependent mechanisms and MR-independent reactive species generation in the pancreatic β-cells [54]. All these mechanisms can explain the association between excess aldosterone, insulin resistance, hyperglycemia and type 2 diabetes.

Regarding clinical evidence, several studies have reported a reduced incidence of type 2 diabetes following treatment with either MR antagonists or adrenalectomy in the setting of PA [55, 56]. Elevated aldosterone levels, even within a normal range, have been associated with the development of insulin resistance in non-diabetic individuals over a 10-year follow-up [57].

On the other hand, from the type 2 diabetes perspective, MR antagonists have demonstrated beneficial effects by improving insulin sensitivity and attenuating kidney disease, underscoring the central role of aldosterone, MR activation and RAAS signalling in metabolic and renal regulation [58]. Supporting this, RAAS activation has been linked with incident type 2 diabetes in adults from the Jackson Heart Study, with adiposity potentially mediating this association, particularly in individuals with prediabetes [59]. Also, aldosterone excess has been found to be prevalent in the setting of type 2 diabetes -even with low renin levels- [60], and associated with diabetic kidney disease development, highlighting the pathological impact of aldosterone in this context [61].

Overall, these findings show that aldosterone can be a key mechanistic driver linking metabolic dysfunction through insulin resistance and type 2 diabetes via MR-dependent and independent pathways including impaired insulin signalling, reduced GLUT4-mediated glucose uptake, pancreatic β-cell dysfunction, oxidative stress and inflammation.

### Aldosterone and dyslipidemia

The association between aldosterone excess and/or PA remains underexplored, with few studies focusing on this relationship. A study including general population found that higher levels of aldosterone were associated with increased serum concentrations of LDL-C, non-HDL-C and decreased HDL-C, and no associations were found with renin and aldosterone-to-renin ratio [62]. Regarding PA, a recent metanalysis has shown that bilateral PA when compared to unilateral PA has worse lipid and metabolic profile with higher values of LDL-C, otal cholesterol, fasting blood glucose, glycated hemoglobin A1c and homeostasis model assessment-insulin resistance (HOMA-IR), with no relevant differences in HDL-C, suggesting that the higher secretion of aldosterone in bilateral PA, is associated with worse lipid profile [63]. Potential mechanisms linking aldosterone with lipid profile disturbances have been studied. In particular, Caveolin-1, which is a scaffolding protein that form caveolae-specialized lipid raft microdomains in the plasma membrane that regulates signals, demonstrated in both in vivo and in vitro models that when its expression is decreased, it activates the effect of aldosterone-MR signalling on lipid pathways [64].

### Aldosterone and hypertension

Aldosterone is a key regulator of blood pressure, through sodium retention and volume expansion, and its excess is a well-known cause of hypertension [65]. Mechanisms linking this association are mainly genomic (ENaC and Na/K ATPase expression) but also related with vascular damage. Excess aldosterone in vascular smooth muscle has been shown to downregulate the IRS-1/AKT signalling pathway, which under normal conditions promotes nitric oxide synthesis. Its downregulation leads to vasoconstriction, increased vascular remodelling and arterial stiffness, ultimately contributing to the development of hypertension. Other mechanisms linking aldosterone with hypertension involve MR and ENaC activation in the brain that mediate central aldosterone-induced sympathetic hyperactivity in hypertension [66].

**Fig. 1:**
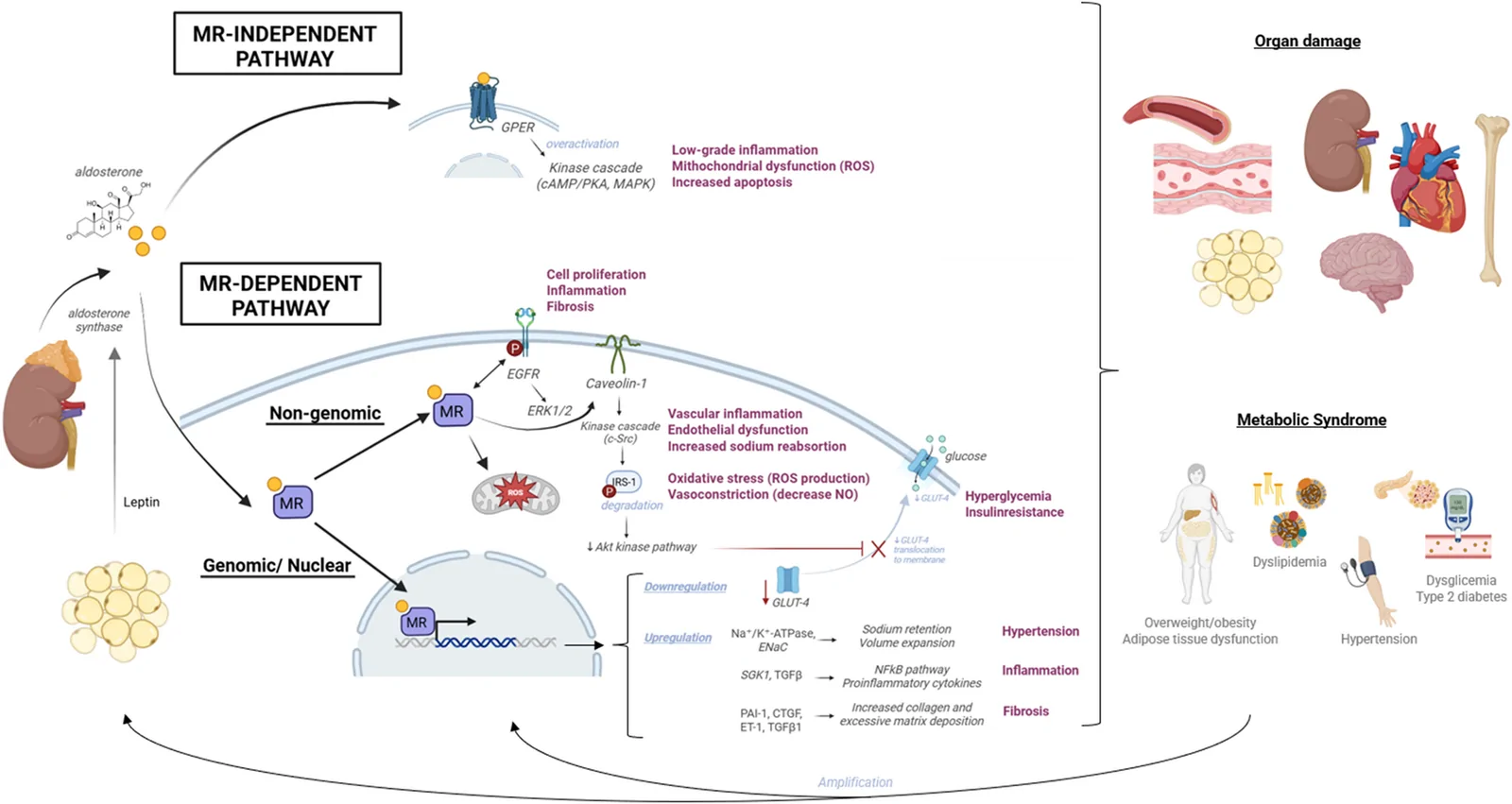
Pathophysiological mechanisms in which aldosterone contributes to metabolic dysfunction and indirectly to MASLD. Aldosterone can be stimulated by the renin–angiotensin system or by other factors, including leptin. Leptin, an adipokine secreted by adipose tissue, induces aldosterone synthase expression, resulting in aldosterone excess. Aldosterone excess can act independently through a MR-independent pathway by overactivating GPER, resulting in mitochondrial dysfunction, production of reactive oxygen species, and inflammation. The main mechanism by which aldosterone acts is through binding to the MR in the cytosol of cells expressing MR. Once aldosterone binds to MR, it can act through a rapid non-genomic pathway, producing mitochondrial dysfunction and ROS formation, and stimulating transmembrane receptor EGFR and transmembrane scaffolding protein Caveolin-1, among others. This can further activate a kinase cascade involving c-Src, leading to endothelial dysfunction, particularly in vascular smooth muscle cells. Additionally, it can induce phosphorylation and degradation of IRS-1, which leads to reduced GLUT-4 translocation to the membrane, resulting in hyperglycemia and insulin resistance. On the other hand, the aldosterone–MR complex can act through a genomic (nuclear) pathway by regulating gene expression. Downregulation of GLUT-4 expression contributes to hyperglycemia and insulin resistance. Upregulation of sodium transporters at the renal level represents one of the classical mechanisms of sodium retention, volume expansion, and hypertension development. Genes regulating inflammatory pathways can be upregulated, leading to inflammation, and specific genes such as PAI-1, CTGF, and ET-1 can directly promote fibrosis. Taken together, these mechanisms can damage different cells and organs, most notably the kidney, heart, and blood vessels. Other consequences of these aldosterone-induced metabolic disturbances include contribution to components of the metabolic syndrome and further amplification of adipose tissue dysfunction, potentially leading to increased leptin and aldosterone synthesis. *Abbreviations*, *ATPase*, adenosine triphosphatase; *cAMP*, cyclic adenosine monophosphate; *c-Src,* cellular Src kinase;* CTGF*, connective tissue growth factor;* EGFR*, epidermal growth factor receptor;* ENaC*, epithelial sodium channel; *ERK1/2,* extracellular signal-regulated kinase *1/2; ET-1*, endothelin-1; GLUT-4, glucose transporter type 4; *GPER*, G protein–coupled estrogen receptor; IRS-1, insulin receptor substrate-1; K⁺, potassium; *MAPK*, mitogen-activated protein kinase; *MR*, mineralocorticoid receptor; *Na⁺*, sodium;* PAI-1*, plasminogen activator inhibitor-1; *PKA*, protein kinase A; *SGK1*, serum- and glucocorticoid-inducible kinase 1; *TGF-β*, transforming growth factor beta

The most common cause of secondary hypertension is PA, which is associated with cardiovascular and renal complications, as well as metabolic disturbances such as insulin resistance and adipose tissue dysfunction [67, 68]. On the other hand, excess aldosterone in primary hypertension has also been linked with worse clinical outcomes. A recent study on individuals with primary hypertension found that aldosterone was an independent factor associated with kidney damage, after adjustment for blood pressure treatment, sex, ethnicity, age, renin levels and many other potential confounding factors [69]. Mechanisms linking hypertension-mediated organ damage associated with MR activation also include non-classical pathways (independent of the sodium-retaining effects) which mediate oxidative stress, inflammation and fibrosis (Fig. 1).

## Crosstalk between aldosterone and MASLD

### Potential mechanistic pathways linking aldosterone and MASLD

**Fig. 2:**
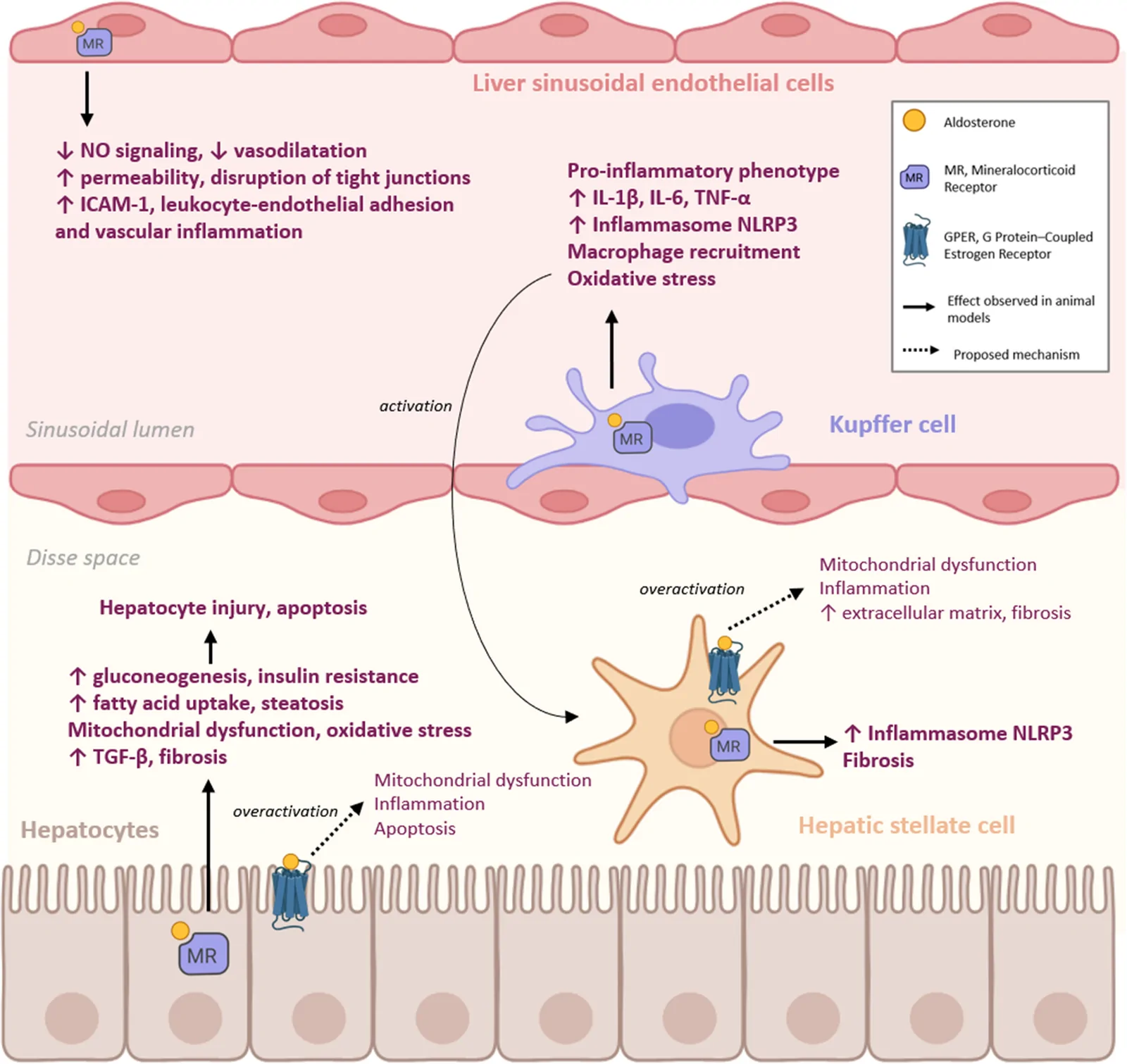
Proposed direct mechanisms of aldosterone on liver cells. MR activation, through cortisol or aldosterone activation, can drive cell-specific pathological responses in the liver that promote MASLD development and progression. In hepatocytes, MR activation disrupts glucose and lipid metabolism, leading to lipid accumulation, oxidative stress, inflammation, and apoptosis. In liver sinusoidal endothelial cells, MR activation induces endothelial dysfunction, capillarization, increased permeability, leukocyte adhesion, and pro-fibrotic signalling. In Kupffer cells, MR activation promotes a pro-inflammatory phenotype with cytokine release, oxidative stress, and immune cell recruitment, further amplifying inflammation and indirectly activating hepatic stellate cells. In hepatic stellate cells, MR signalling contributes to activation into collagen-producing myofibroblast-like cells, driving fibrosis, although MR-independent effects of aldosterone could be also implicated. Additionally, aldosterone can act through MR-independent pathways, particularly via GPER, which is expressed in hepatocytes and stellate cells and may contribute to liver injury, although its pathological role remains incompletely defined. *Abbreviations,* *ICAM-1*, intercellular adhesion molecule-1;* GPER*, *G* protein–coupled estrogen receptor; *MR*, mineralocorticoid receptor; *TNF-α*, tumour necrosis factor alpha; *IL*, interleukin; *NLRP,* *NOD-like* receptor pyrin domain–containing inflammasome;* LSEC*, liver sinusoidal endothelial cells; *NO*, nitric oxide

The association between aldosterone and MASLD is supported by the evidence linking aldosterone to each component of the metabolic syndrome as discussed in the previous section. These mechanisms can contribute to MASLD in an indirect pathway and have been stablished in previous clinical and animal models (Fig. 1). However, beyond these indirect pathways, excess aldosterone may also exert a direct role in MASLD via its effects on inflammation, oxidative stress and fibrosis, through the proposed mechanisms discussed below (Fig. 2). The MR has been found to be expressed in different hepatic cells including the hepatocytes, liver sinusoidal endothelial cells (LSEC), Kupffer cells and hepatic stellate cells [70]. Nevertheless, information on the mechanistic pathways between aldosterone and MASLD specific for every liver cell type is scarce.

In the natural course of MASLD, early stages such as steatosis and steatohepatitis can be linked to aldosterone via inflammatory pathways and metabolic dysregulation. Progression to fibrosis is often amplified by other determinants in addition to metabolic syndrome and adipose tissue dysfunction, which include diet, alcohol intake, endocrine chemical disruptors and genetic predisposition []. However, the mechanisms by which aldosterone may influence fibrotic progression in MASLD have not been elucidated.

In hepatocytes from rats, increased MR activation is associated with dysregulation of glucose and lipid metabolism, leading to increased apoptosis and a proinflammatory environment [71, 72]. These disturbances can impair hepatocellular lipid metabolism, affecting the primary mechanisms underlying hepatic steatosis, the earliest stage of MASLD. In hepatic steatosis, hepatocytes accumulate lipid droplets, which in excess lead to lipotoxicity, thereby amplifying oxidative stress and inflammation, and ultimately triggering hepatocyte injury and apoptosis [73].

In response to the metabolic stress from the hepatocytes, LSEC undergo capillarization and lose their anti-inflammatory and anti-fibrotic properties, which include prevention of Kupffer cell activation, immune cell recruitment, production of vasodilators and maintenance of hepatic stellate cell quiescence, among others. This leads to further hepatocyte dysfunction, and secretion of pro-fibrotic mediators (e.g., TGF-β) from LSEC [74, 75]. In this regard, TGF-β is one of the proteins whose expression is upregulated by excess of aldosterone via its genomic action, suggesting that a crosstalk between aldosterone excess and LSEC may contribute to the development of liver fibrosis. Activation of the MR in LSEC can lead to endothelial dysfunction through nitric oxide signalling dysregulation, increased permeability and leukocyte adhesion through upregulation of intracellular adhesion molecule-1 (ICAM-1), contributing to inflammation and fibrosis [76, 77].

Injured hepatocytes and LSEC can activate Kupffer cells and hepatic stellate cells, which are key mediators in liver fibrosis. Kupffer cells are the resident macrophages of the liver, which release proinflammatory cytokines, amplifying hepatic inflammation and further inducing the activation of hepatic stellate cells [74]. Activation of the MR in Kupffer cells can lead to CD8 T-cell activation, a polarization towards a pro-inflammatory phenotype, cytokine production (IL-1β, IL-6, TNF-α), oxidative stress, macrophage recruitment and indirect stellate cell activation through inflammatory mediators [78, 79].

Activated hepatic stellate cells can differentiate into myofibroblast-like cells, producing collagen and extracellular matrix, leading to hepatic fibrosis and architectural distortion. Regarding MR expression in the hepatic stellate cells, contradictory results have been found in rats, with some studies detecting mRNA [80, 81]. However, one study did not find evidence of MR mRNA but observed an increase of extracellular matrix proteins after aldosterone administration [82], suggesting additional MR-independent mechanisms of aldosterone harm in the liver [83]. Aldosterone, through MR activation in Kupffer cells and hepatic stellate cells, promotes NLRP3 inflammasome expression and assembly that further activates hepatic stellate cells and drives fibrosis in mice, which has been found to be a key driver of fibrosis in preclinical models [24, 84].

Besides MR activation, aldosterone can act through MR-independent mechanisms in the liver that could explain the mentioned findings and the associations found in the studies below, especially since MR in the liver can be also activated by cortisol due to low 11β-HSD2 local expression. The main described MR-independent mechanism in animal models is through GPER activation, which also has been found to be expressed in liver cells from animal models -mainly hepatocytes and stellate cells- [85–87]. Studies of GPER on liver have been focused mainly on its physiological protective estrogen-activation, and information on harmful consequences of its overactivation in the liver through aldosterone excess is lacking. More information is needed on MR-independent mechanisms of aldosterone, especially in MASLD. 

### Clinical and experimental studies on aldosterone and MASLD

Most of the available evidence derives from animal models or human individuals with PA (Table 1). Regarding PA, a cross-sectional study including 120 individuals showed that plasma aldosterone levels were associated with the presence of MASLD in PA [88], whereas another cross-sectional single centre study on 91 patients showed that liver steatosis was increased at PA diagnosis, and improved after medical or surgical treatment [89]. Improvements on liver steatosis after PA treatment could be due to broader metabolic disturbances related to aldosterone. A third study comparing 222 individuals with and 222 without PA showed that MASLD was more common in PA, where hypokalemic PA individuals had the worse metabolic status (more severe dyslipidemia, insulin resistance and increased serum uric acid levels) [90]. These studies are mainly cross sectional, limiting the assessment of causality, and mainly include individuals with PA in whom additional confounding factors such as the severity of the metabolic diseases and mild autonomous cortisol co-secretion could be present.

**Table 1:** Main studies on aldosterone and MASLD

First Author, year	Type of research	Type of study, sample size, or type of intervention	Main results
Aldosterone and MASLD associations			
Tizianel I, 2025(89)	Observational, clinical data	Cross-sectional study including 149 individuals. Among them 41 with PA (20 unilateral, 21 bilateral), 50 with non-functioning adenomas, 48 MACS and 10 adrenal CS.	Prevalence of MASLD assessed by liver/spleen ratio* was higher in PA compared to non-functioning adenomas (49% vs. 14%, *p* < 0.001) and MACS (49% vs. 25%, *p* < 0.05) and similar to adrenal CS (49% vs. 45%, *p* = 0.61).
Zeng Q, 2025(91)	Observational, clinical data	Mendelian randomisation study of 125,357 participants from the UK Biobank.	Renin-independent-aldosteronism assessed through polygenic risk scores was genetically associated with higher risks of MASLD (OR 1.28, 95% CI 1.09–1.49) and cirrhosis (OR 1.49, 95%CI 1.03–2.16).
Hu J,2023(92)	Observational, clinical data	Cohort study of 3,713 hypertensive individuals over a 10-year retrospective period.	There was a non-linear positive J-shaped relationship between plasma aldosterone and new-onset MASLD. In particular plasma aldosterone increased risk of MASLD with a HR for highest tertile 3 of 1.71 (95%CI 1.47–1.98), and an inflection point in plasma aldosterone at 13ng/dL for the non-linear association.
Chen Y, 2021(90)	Observational, clinical data	Case-control study including 444 individuals (222 with PA and 222 without).	The prevalence of MASLD was higher in PA patients than in non-PA patients (35.1% vs. 29.7%), although the patterns of obesity, dyslipidemia and insulin resistance were similar. Within PA, hypokalemic PA had a more MASLD prevalence (44% vs. 27%, *p* < 0.05) and worse metabolic status than normokalemic PA patients, including higher BMI (25.4 vs. 24.1, *p* < 0.05),
Kumar A, 2022(93)	Observational, clinical data	Cross-sectional community-based study of 2,507 African Americans from the Jackson Heart Study who were not under ACEi, ARB, or MR antagonists.	Liver fat through CT scan liver attenuation as measured and each doubling of aldosterone was associated with 1.08 HU decrease (95% CI 1.47 - -0.69, *p* < 0.001), which was significant only in women after full multivariate adjustment by decreasing 0.94 HU (95% CI − 1.35 - −0.52, *p* < 0.001).
Fallo F, 2010(88)	Observational, clinical data	Case-control study matched by sex, age and BMI including 120 individuals (40 PA, 40 with low-renin essential hypertension and 40 normotensive).	MASLD was a frequent finding in PA (57.5%), where PA with hypokalemia were more insulin resistant compared with normokalemia (HOMA index 5.1 vs. 4.3, *p* < 0.01) and had higher prevalence of MASLD 72.7% vs. 38.8%, *p* < 0.05).
Mineralocorticoid receptor antagonism/defficiency			
Gamliel-Lazarovich A, 2013(94)	Experimental, preclinical animal models	Mice in high-fat diet, treated with adrenalectomy or eplerenone	Neither adrenalectomy nor eplerenone affected fatty liver formation or body weight. In cultured hepatocytes, triglyceride loading induced by high glucose, oleic acid or very low-density lipoprotein was not affected by aldosterone, spironolactone or eplerenone.
Muñoz-Durango N, 2020(79)	Experimental, preclinical animal models	Mice with methionine-choline deficient diet-induced MASH with deletion in MR from myeloid cells vs. controls.	Mice with MRs deletion exhibited reduced hepatic inflammation and lower hepatic triglyceride content; as well as costimulatory molecule CD86 by dendritic cells and the CD25 activation marker in CD8 + T cells which were significantly reduced. Loss of MR in myeloid cells reduces lipid accumulation in the liver, in part through modulating the adaptive immune response, which is pivotal for the development of steatosis.

*Liver/spleen ratio refers to the ratio of hepatic attenuation to splenic attenuation measured in CT scan with Hounsfield units. Low values indicate hepatic steatosis

Abbreviations: ACEi, angiotensin-converting enzyme inhibitor*; ARB, angiotensin II receptor blocker; BMI*, body mass index; *CD*, cluster of differentiation; *CI*, Confidence Interval; *CS*, Cushing Syndrome; *CT*, Computed Tomography; *HOMA*, homeostasis model assessment; *HU*, Hounsfield Units; *HR*, Hazard Ratio; PA, primary aldosteronism; *MACS*, mild autonomous cortisol secretion; *MASLD*, metabolic dysfunction–associated steatotic liver disease; MASH, metabolic dysfunction-associated steatohepatitis; *MR*, mineralocorticoid receptor; *OR*, Odds Ratio; *PA*, primary aldosteronism; *UK*, United Kingdom

A broader genetic-association study including 125,357 individuals from the UK biobank, showed through a Mendelian randomisation analysis that increased polygenic risk scores for renin-independent aldosteronism (a broader category which may include individuals with PA) were associated with increased risk of MASLD and MASLD-related cirrhosis [91]. By using genetic proxies for aldosterone dysregulation, Mendelian randomisation reduces confounding and provides support for a causal and mechanistic role of aldosterone in MASLD development and progression.

Besides PA, a cohort study including 3,713 hypertensive individuals over a 10 year period, showed that increased aldosterone plasma concentrations were associated with new-onset MASLD, suggesting a causal link [92]. Another study including 2,507 individuals from the Jackson Heart Study cohort found that aldosterone levels were significantly correlated with increased hepatic steatosis measured through with liver attenuation in computed tomography, an association that only remained in women after fully-adjusted multivariable models, despite being a cross-sectional study which limits causal inference [93].

Animal models have shown that in mice, aldosterone levels could be involved in diet-induced insulin resistance and glucose disturbances [79]. However, one study by Gamliel-Lazarovich A, et al. found no association in the development of fatty liver disease [94]. Regarding this study, consideration should be taken since the authors compared mice treated with eplerenone with mice who underwent bilateral adrenalectomy and required saline solution for survival, so this could affect the interpretation of the results. Additionally, differences in animal models exist across studies, with mice being exposed to different diets and interventions (Table 1).

### Evidence on RAAS and MR blockade in MASLD

Understanding the role of aldosterone in MASLD is relevant not only for advancing knowledge of disease pathophysiology, but also for identifying potential therapeutic targets. MR antagonists have demonstrated beneficial effects on cardiac function and renal disease, including within the cardio–kidney–metabolic syndrome, primarily through attenuation of MR-mediated inflammation, oxidative stress, fibrosis, and sodium retention, as well as improvement of insulin sensitivity. In line with this, prior evidence on RAAS blockade using ACEi and ARB indicates that individuals with MASLD receiving ACEi/ARB therapy exhibit a reduced risk of MASLD progression, adverse liver outcomes and cardiovascular events [25, 95–98]. However, these results could be a reflection of RAAS modulation rather than specific to aldosterone blockade.

MR antagonists have a direct effect on aldosterone levels but also could influence other molecules that bind to MR such as cortisol and its related-consequences. Importantly, the drug effects do not suppress aldosterone itself, allowing persistently elevated hormone levels to potentially exert MR-independent effects leading to oxidative stress and a proinflammatory environment. Current available MR antagonists include spironolactone, eplerenone and finerenone. When starting treatment with MR antagonists, patients should be monitored for hyperkalemia -especially in the setting of declining renal function-, with dose adjustments as appropriate. Spirinolactone is commonly used in cirrhosis with ascites, although careful electrolyte monitoring is required to prevent neurological complications.

Spironolactone and eplerenone are steroidal MR antagonists, where spironolactone is less selective and binds to androgen and progesterone receptors, resulting in an increased hormonal side effects such as gynecomastia. Eplerenone is selective for the MR and has less adverse effects. Finerenone is a nonsteroidal MR antagonist, with high specificity for the MR, not interacting with other steroid receptors and having less side effects. In contrast to steroidal MR antagonists, finerenone shows less distribution to renal epithelial tissues, as its structure limits penetration across epithelial barriers, decreasing the risk of hyperkalemia.

Spironolactone, the most used MR blocker, has shown in a cohort study of 7,241 hypertensive individuals to have a benefit by reducing the risk of MASLD development [99]. Additionally, an interventional study combining spironolactone with vitamin E showed that in MASLD the combination offered an improvement on insulin resistance parameters when compared to vitamin E alone [100]. Finerenone, a relevant MR antagonist aimed at improving diabetic kidney disease, showed in a subgroup analysis of the FIDELITY study, relevant improvement of kidney outcomes, as well as cardiovascular benefits on individuals with increased liver fibrosis risk assessed through FIB-4 index [101]. Information on other non-invasive liver tests, and their changes after treatment with MR antagonists is lacking. On the other hand, a single open-label proof-of-concept study with eplerenone on individuals with human immunodeficiency virus infection and hepatic steatosis showed an early increase of liver fat, although there was no control group [102]. Notable heterogeneity exists across the mentioned studies in terms of study population characteristics, liver disease measurements and outcomes, warranting careful interpretation. However, current MASLD guidelines do not incorporate this recommendation for the treatment due to the lack of RCT specifically designed for this purpose.

### Sex-differences in the association between aldosterone and MASLD

Aldosterone and renin levels tend to be increased in men when compared to premenopausal women. Reference values for renin and aldosterone can differ also by ethnicity, where in Caucasian population range values for women have been described (renin 6.1-62.7 mIU/L, aldosterone < 138-1179 pmol/L) and men (renin 6.1-62.7 mIU/L, aldosterone < 138-1179pmol/L) [103]. Despite women exhibit lower values, they present increased adrenal responsiveness, resulting in an enhanced aldosterone response to acute stimulation, which can manifest as greater salt sensitivity in blood pressure regulation [104]. Special attention should be placed since aldosterone and renin levels can fluctuate across the menstrual cycle in premenopausal women, with the luteal phase increasing levels of both hormones when comparing to the follicular phase, which can be due to increased levels of progesterone that antagonise the MR [105, 106].

Estrogens can modulate the MR pathway, inhibiting aldosterone synthesis via the estrogen receptor-β, resulting in lower aldosterone levels and blood pressure in premenopausal women. After the menopause, when estradiol levels decline, the estrogen-mediated inhibition is lost, and GPER receptors are activated stimulating aldosterone biosynthesis and a positive feedback loop, ultimately increasing blood pressure [107].

The impact of aldosterone in different tissues can present differences by sex, where there is evidence that the impact of aldosterone on cardiac remodelling is more pronounced in women independent of menopausal status [108]. Regarding adipose tissue dysfunction, leptin secretion triggers aldosterone synthase expression and increased plasma aldosterone, especially in female animal models. Additionally, in female animals, aldosterone blockade with spironolactone restores blood pressure [35]. Male animals, however, exhibit enhanced sympathetic responses to leptin when compared to female [35, 109]. Body fat distribution is also associated with aldosterone sex-related differences, where evidence in humans has demonstrated that aldosterone in men has a stronger association with ectopic cardiac adiposity, whilst in women the association is stronger with total body fat [110]. It is particularly relevant when assessing menopausal status, as estrogens exert a protective effect by reducing VAT; an effect that disappears after menopause leading to increased cardiometabolic risk.

Regarding whether there may be a different response by sex to treatment with MR antagonists, a post-hoc subgroup analysis of the FIDELIO trial with finerenone, showed no differences in the risk in cardiovascular and kidney outcomes after stratifying by sex and menopausal status, including evaluation of interaction [111]. However, a more pronounced reduction in heart failure hospitalisations were observed in men [111].

There are also sex differences in MASLD, where premenopausal women benefit from the protective effects of estrogens on hepatic lipid metabolism, inflammation and fibrogenesis, when compared to men in which androgens impact negatively presenting increased prevalence and severity of MASLD. After menopause, estrogen deficiency increases the susceptibility of MASLD and accelerates fibrosis progression [112–114]. The interplay between sex hormones and genetic variants (e.g., PNPLA3, TM6SF2) are key in MASLD development and disease progression [112].

Given the known sex differences in aldosterone, leptin-induced aldosterone excess, and MASLD, we may think that the protective effect of estrogens over MASLD in premenopausal women could be associated with lower aldosterone levels when compared to men. In postmenopausal women, body fat distribution and leptin-triggered aldosterone enhancement could influence the development and progression of MASLD. However, sex differences in aldosterone and MASLD remains a field for future research.

## Future perspectives

### New and future treatments on aldosterone and its potential impact on MASLD

Recent advances in aldosterone-targeting therapies include non-steroidal-MR antagonists and aldosterone synthase inhibitors. Among non-steroidal MR antagonists, finerenone is the most implemented agent, which acts as a selective non-steroidal MR antagonist inducing changes in the receptor that reduce recruitment of transcriptional cofactors attenuating proinflammatory and profibrotic gene expression. Finerenone is approved for individuals with diabetic kidney disease and has demonstrated improvement in kidney and cardiovascular outcomes with a superior safety profile when compared to steroidal MR antagnoists [115]. Esaxerenone is another non-steroidal MR antagonist, which is commercialised in some countries for PA, hypertension and diabetic kidney disease and has shown to reduce blood pressure and albuminuria, with a renoprotective effect that could be partly independent of blood pressure lowering [116]. Ocedurenone represents a newer non-steroidal-MR antagonist under development, which is highly selective to MR to minimise adverse effects, designed for advanced chronic kidney disease and resistant hypertension, showing favorable results in phase 2 clinical trials [117].

Aldosterone synthase inhibitors constitute an emerging drug class designed to directly inhibit aldosterone synthase, the enzyme involved in aldosterone biosynthesis, resulting in reduced aldosterone production and attenuation of its pathological effects. By selectively targeting aldosterone synthase instead of the MR, this emerging drug class improves pharmacological specificity and reduces the impact on other molecules that bind the MR such as cortisol. Additionally, it can benefit from decreasing the overactivation of MR-independent pathways which are not triggered with MR antagonists. This is especially relevant given the growing evidence of MR-independent pathways in which aldosterone excess could have a detrimental metabolic effect, such as the one mediated through GPER overactivation (Fig. 1).

Novel agents in the aldosterone synthase inhibitors category include baxdrostat, lorundrostat, dexfadrostat phosphate, and vicardostat, which have reported promising results in phase 2 and phase 3 trials regarding blood pressure reductions and kidney damage parameters [118–121]. Baxdrostat is the most advanced compound in this class, which has been primarily studied in patients with resistant or uncontrolled hypertension and is being explored in PA, showing dose-dependent reductions in aldosterone levels and blood pressure lowering along with low rates of hyperkalemia and good tolerability [18]. Other aldosterone synthase inhibitors including larundrostat, dexfadrostat and vicadrostat are in earlier stages of development. These novel agents are under active investigation for broader indications and may soon redefine the therapeutic landscape for aldosterone-mediated diseases.

Future studies will be needed to clarify the potential impact of selective aldosterone-synthase inhibitors on the development and progression of MASLD.

### The role of the RAAS in precision medicine for obesity and MASLD

While aldosterone can play a key independent role in metabolic dysfunction, it can also be the final effector of the RAAS cascade. Thus, understanding the RAAS system is essential for a holistic view of the hormonal, metabolic and inflammatory pathways involved in obesity and MASLD.

In the setting of adipose tissue dysfunction and MASLD, overactivation of the RAAS axis promotes hepatic steatosis, inflammation, insulin resistance and fibrosis, through the previously mentioned multiple mechanistic pathways. Obesity and adipose tissue dysfunction could be playing an important role as triggers of aldosterone excess in the context of MASLD, so the assessment of MASLD and RAAS should also focus on weight loss with consequent aldosterone reduction [37, 38].

Refining the assessment of the RAAS axis and its regulatory mechanisms in clinical practice could improve patient stratification and inform personalised treatment decisions, with the aim to improve metabolic dysfunction and prevent the development and progression of comorbid conditions such as MASLD. In particular, evaluating circulating RAAS components, including renin and aldosterone, in metabolically unhealthy individuals, may aid the identification of candidates for targeted pharmacological intervention, constituting a potential important step towards precision medicine. Future metabolomic profiling may help in identifying intermediate metabolites within the RAAS pathway that contribute to aldosterone and RAAS regulation, ultimately enabling risk stratification based on metabolomic signatures. Including additional evaluation on sex-related hormonal and menopausal status could help further identify patients at higher risk of metabolic and organ damage associated to MASLD and increased aldosterone/RAAS activation.

### Clinical validation framework for assessing the link between aldosterone and MASLD

To enhance the translational relevance of the evidence summarised in the manuscript, we suggest to incorporate the following measures in the MASLD clinical assessment beyond the standard evaluation:

A) Anthropometric measurements: waist circumference, waist-to-height ratio. If available: bioimpedance analysis, dual-energy X-ray absorptiometry, fat and muscle segmentation through computed tomography, functional muscle strength test (e.g., handgrip strenght).
B) Adipose tissue and metabolic dysfunction biomarkers: HDL-cholesterol, LDL-cholesterol, Triglycerides, TG/HDL ratio, Triglyceride-Glucose index, leptin, adiponectin, leptin/adiponectin ratio,
C) RAAS molecules: plasma aldosterone, renin (direct renin or plasma renin activity), aldosterone-renin-ratio.
D) MASLD outcomes: Fibrosis-4 index, Liver Risk Score, Enhanced Liver Fibrosis test (ELF, when available), Hepamet fibrosis score, transient elastography including the controlled attenuation parameter for steatosis assessment and liver stiffness for fibrosis.

Based on this integrated assessment, patients could be stratified according to fibrosis risk, prioritizing more intensive evaluation and closer follow-up in those with higher-risk profiles. Stratification may also incorporate aldosterone levels and the aldosterone-to-renin ratio, although these should be interpreted with caution in patients receiving medications that affect RAAS. In addition, the degree of metabolic dysfunction -captured by lipid-derived ratios and adipokine profiles- should be considered to better reflect the systemic drivers of disease progression. Finally, anthropometric measures that more accurately represent body fat distribution, and particularly visceral adiposity, should be included given their mechanistic link with aldosterone dysregulation and MASLD severity.

### Future research priorities in MASLD and aldosterone

Despite growing evidence linking aldosterone to metabolic dysfunction and MASLD, several important knowledge gaps and areas of ongoing debate remain.

The contribution of aldosterone to adipose tissue dysfunction requires further clarification, particularly regarding adipose tissue depots, body fat distribution, and cell-specific MR activity.

At the hepatic level, the cell-type–specific effects of aldosterone across hepatocytes, hepatic stellate cells, Kupffer cells, and LSEC warrant further investigation.

Potential modifiers, including ethnicity and cardiorenometabolic comorbidities, have been variably addressed across studies and represent important factors for the development of personalized medicine approaches. Confounding by glucocorticoid receptor activation, such as mild autonomous cortisol secretion, may also influence observed associations between aldosterone, insulin resistance, and adipose tissue dysfunction and deserves greater consideration.

Sex-specific differences influencing susceptibility and disease progression are increasingly recognized but remain incompletely characterized.

Clinical evidence is limited by relatively short follow-up and heterogeneous study designs, contributing to some variability in findings and uncertainty regarding causality versus association.

Future research should build on existing evidence through large prospective studies and mechanistic investigations in humans, alongside the development of biomarkers of aldosterone exposure, MR activity, and downstream metabolic effects, to support a more integrated RAAS assessment in MASLD within the broader cardiorenometabolic disease framework.

## Data Availability

No datasets were generated or analysed during the current study.

## References

[1] Huang DQ, Wong VWS, Rinella ME, Boursier J, Lazarus JV, Yki-Järvinen H, et al. Metabolic dysfunction-associated steatotic liver disease in adults. Nat Rev Dis Prim. 2025;11(1):14. 10.1038/s41572-025-00599-1 40050362

[2] Israelsen M, Francque S, Tsochatzis EA, Krag A. Steatotic liver disease. Lancet (London England). 2024;404(10464):1761–78. 10.1016/S0140-6736(24)01811-7 39488409

[3] Kuchay MS, Choudhary NS, Ramos-Molina B. Pathophysiological underpinnings of metabolic dysfunction-associated steatotic liver disease. Am J Physiol Cell Physiol. 2025;328(5):C1637–66. 10.1152/ajpcell.00951.2024 40244183

[4] Targher G, Valenti L, Byrne CD. Metabolic Dysfunction-Associated Steatotic Liver Disease. N Engl J Med. 2025;393(7):683–98. 10.1056/NEJMra2412865 40802944

[5] Yang M, Ma X, Xuan X, Deng H, Chen Q, Yuan L. Liraglutide Attenuates Non-Alcoholic Fatty Liver Disease in Mice by Regulating the Local Renin-Angiotensin System. Front Pharmacol. 2020;11:432. 10.3389/fphar.2020.00432 PMC7156971 32322207

[6] Lu P, Liu H, Yin H, Yang L. Expression of angiotensinogen during hepatic fibrogenesis and its effect on hepatic stellate cells. Med Sci Monit Int Med J Exp Clin Res. 2011;17(9):BR248–56. 10.12659/MSM.881928 PMC3560510 21873937

[7] Hurr C, Simonyan H, Morgan DA, Rahmouni K, Young CN. Liver sympathetic denervation reverses obesity-induced hepatic steatosis. J Physiol. 2019;597(17):4565–80. 10.1113/JP277994 PMC6716997 31278754

[8] Carnagarin R, Tan K, Adams L, Matthews VB, Kiuchi MG, Marisol Lugo Gavidia L et al. Metabolic Dysfunction-Associated Fatty Liver Disease (MAFLD)-A Condition Associated with Heightened Sympathetic Activation. Int J Mol Sci. 2021;22(8). 10.3390/ijms22084241 PMC8073135 33921881

[9] Kahlon T, Carlisle S, Otero Mostacero D, Williams N, Trainor P, DeFilippis AP, Angiotensinogen. More Than its Downstream Products: Evidence From Population Studies and Novel Therapeutics. JACC Heart Fail. 2022;10(10):699–713. 10.1016/j.jchf.2022.06.005 35963818

[10] Kanbay M, Guldan M, Ozbek L, Al-Shiab R, Laffin LJ. Dallas, Tex. Angiotensinogen Reconsidered: Evolving Perspectives in Hypertension Research. Hypertens (1979). 2026. 10.1161/HYPERTENSIONAHA.125.26091 41853854

[11] Moreira de Macêdo S, Guimarães TA, Feltenberger JD, Sousa Santos SH. The role of renin-angiotensin system modulation on treatment and prevention of liver diseases. Peptides. 2014;62:189–96. 10.1016/j.peptides.2014.10.005 25453980

[12] Wu Y, Ma KL, Zhang Y, Wen Y, Wang GH, Hu ZB, et al. Lipid disorder and intrahepatic renin-angiotensin system activation synergistically contribute to non-alcoholic fatty liver disease. Liver Int Off J Int Assoc Study Liver. 2016;36(10):1525–34. 10.1111/liv.13131 27028410

[13] Lai X, Yang L, Légaré S, Angileri F, Chen X, Fang Q, et al. Dose-response relationship between serum uric acid levels and risk of incident coronary heart disease in the Dongfeng-Tongji Cohort. Int J Cardiol. 2016;224:299–304. 10.1016/j.ijcard.2016.09.035 27665401

[14] McGrath MS, Wentworth BJ. The Renin-Angiotensin System in Liver Disease. Int J Mol Sci. 2024;25(11). 10.3390/ijms25115807 PMC11172481 38891995

[15] Bollag WB. Regulation of aldosterone synthesis and secretion. Compr Physiol. 2014;4(3):1017–55. 10.1002/cphy.c130037 24944029

[16] Funder JW. The nongenomic actions of aldosterone. Endocr Rev. 2005;26(3):313–21. 10.1210/er.2005-0004 15814845

[17] Goupil R, Desbiens L-C, Merabtine A, Agharazii M, Madore F, Vaidya A, et al. Subclinical Primary Aldosteronism and Major Adverse Cardiovascular Events: A Longitudinal Population-Based Cohort Study. Circulation. 2025;152(13):913–23. 10.1161/CIRCULATIONAHA.124.073507 PMC12243960 40631720

[18] Flack JM, Azizi M, Brown JM, Dwyer JP, Fronczek J, Jones ESW, et al. Efficacy and Safety of Baxdrostat in Uncontrolled and Resistant Hypertension. N Engl J Med. 2025;393(14):1363–74. 10.1056/NEJMoa2507109 PMC7618089 40888730

[19] Fioretti F, Testani JM, Tio MC, Pitt B, Butler J. Aldosterone and Aldosterone Modulation in Cardio-Kidney Diseases. J Am Coll Cardiol. 2025;86(5):354–73. 10.1016/j.jacc.2025.06.012 40738563

[20] Ndumele CE, Rangaswami J, Chow SL, Neeland IJ, Tuttle KR, Khan SS, et al. Cardiovascular-Kidney-Metabolic Health: A Presidential Advisory From the American Heart Association. Circulation. 2023;148(20):1606–35. 10.1161/CIR.0000000000001184 37807924

[21] Bochud M, Nussberger J, Bovet P, Maillard MR, Elston RC, Paccaud F et al. Dallas, Tex. Plasma aldosterone is independently associated with the metabolic syndrome. Hypertens (1979). 2006;48(2):239–45. 10.1161/01.HYP.0000231338.41548.fc 16785327

[22] Bothou C, Beuschlein F, Spyroglou A. Links between aldosterone excess and metabolic complications: A comprehensive review. Diabetes Metab. 2020;46(1):1–7. 10.1016/j.diabet.2019.02.003 30825519

[23] Gries JJ, Lazarus JV, Brennan PN, Siddiqui MS, Targher G, Lang CC, et al. Interdisciplinary perspectives on the co-management of metabolic dysfunction-associated steatotic liver disease and coronary artery disease. lancet Gastroenterol Hepatol. 2025;10(1):82–94. 10.1016/S2468-1253(24)00310-8 39674228

[24] Li Y, Zhang Y, Chen T, Huang Y, Zhang Y, Geng S, et al. Role of aldosterone in the activation of primary mice hepatic stellate cell and liver fibrosis via NLRP3 inflammasome. J Gastroenterol Hepatol. 2020;35(6):1069–77. 10.1111/jgh.14961 31860730

[25] Kuang X, Naiki-Ito A, Kawamura H, Murakami A, Komura M, Kato H, et al. Hepatic stellate cell inhibition by angiotensin II receptor blocker mitigates liver injury and fibrosis via NF-κB-galectin-3 suppression in a rat nonalcoholic steatohepatitis model. Arch Toxicol. 2025;99(8):3393–412. 10.1007/s00204-025-04075-3 40488883

[26] Huang Z, Khalifa MO, Li P, Huang Y, Gu W, Li T-S. Angiotensin receptor blocker alleviates liver fibrosis by altering the mechanotransduction properties of hepatic stellate cells. Am J Physiol Gastrointest Liver Physiol. 2022;322(4):G446–56. 10.1152/ajpgi.00238.2021 35138187

[27] Hermidorff MM, de Assis LVM, Isoldi MC. Genomic and rapid effects of aldosterone: what we know and do not know thus far. Heart Fail Rev. 2017;22(1):65–89. 10.1007/s10741-016-9591-2 27942913

[28] Dooley R, Harvey BJ, Thomas W. Non-genomic actions of aldosterone: from receptors and signals to membrane targets. Mol Cell Endocrinol. 2012;350(2):223–34. 10.1016/j.mce.2011.07.019 21801805

[29] Huby A-C, Antonova G, Groenendyk J, Gomez-Sanchez CE, Bollag WB, Filosa JA, et al. Adipocyte-Derived Hormone Leptin Is a Direct Regulator of Aldosterone Secretion, Which Promotes Endothelial Dysfunction and Cardiac Fibrosis. Circulation. 2015;132(22):2134–45. 10.1161/CIRCULATIONAHA.115.018226 26362633

[30] Faulkner JL, Bruder-Nascimento T, Belin de Chantemèle EJ. The regulation of aldosterone secretion by leptin: implications in obesity-related cardiovascular disease. Curr Opin Nephrol Hypertens. 2018;27(2):63–9. 10.1097/MNH.0000000000000384 PMC6053669 29135585

[31] Jing F, Mogi M, Horiuchi M. Role of renin-angiotensin-aldosterone system in adipose tissue dysfunction. Mol Cell Endocrinol. 2013;378(1–2):23–8. 10.1016/j.mce.2012.03.005 22465098

[32] Briones AM, Nguyen Dinh Cat A, Callera GE, Yogi A, Burger D, He Y et al. Adipocytes produce aldosterone through calcineurin-dependent signaling pathways: implications in diabetes mellitus-associated obesity and vascular dysfunction. Hypertens (Dallas, Tex 1979). 2012;59(5):1069–78. 10.1161/HYPERTENSIONAHA.111.190223 22493070

[33] Dinh Cat AN, Friederich-Persson M, White A, Touyz RM. Adipocytes, aldosterone and obesity-related hypertension. J Mol Endocrinol. 2016;57(1):F7–21. 10.1530/JME-16-0025 27357931

[34] Ehrhart-Bornstein M, Arakelyan K, Krug AW, Scherbaum WA, Bornstein SR. Fat cells may be the obesity-hypertension link: human adipogenic factors stimulate aldosterone secretion from adrenocortical cells. Endocr Res. 2004;30(4):865–70. 10.1081/erc-200044122 15666838

[35] Huby A-C, Otvos LJ, Belin de Chantemèle EJ. Leptin Induces Hypertension and Endothelial Dysfunction via Aldosterone-Dependent Mechanisms in Obese Female Mice. Hypertens (Dallas Tex 1979). 2016;67(5):1020–8. 10.1161/HYPERTENSIONAHA.115.06642 PMC5088432 26953321

[36] Ling G, Bruno J, Albert SG, Dhindsa S. Fatty acids as a direct regulator of aldosterone hypersecretion. Mol Cell Endocrinol. 2023;561:111836. 10.1016/j.mce.2022.111836 36549461

[37] Berney M, Vakilzadeh N, Maillard M, Faouzi M, Grouzmann E, Bonny O, et al. Bariatric Surgery Induces a Differential Effect on Plasma Aldosterone in Comparison to Dietary Advice Alone. Front Endocrinol (Lausanne). 2021;12:745045. 10.3389/fendo.2021.745045 PMC8525894 34675881

[38] Cooper JN, Fried L, Tepper P, Barinas-Mitchell E, Conroy MB, Evans RW, et al. Changes in serum aldosterone are associated with changes in obesity-related factors in normotensive overweight and obese young adults. Hypertens Res. 2013;36(10):895–901. 10.1038/hr.2013.45 PMC3766434 23657296

[39] Oliveras A, Galceran I, Goday A, Vázquez S, Sans L, Riera M et al. Improvement of Arterial Stiffness One Month after Bariatric Surgery and Potential Mechanisms. J Clin Med. 2021;10(4). 10.3390/jcm10040691 PMC7916665 33578924

[40] Oliveras A, Molina L, Goday A, Sans L, Riera M, Vazquez S, et al. Effect of bariatric surgery on cardiac structure and function in obese patients: Role of the renin-angiotensin system. J Clin Hypertens (Greenwich). 2021;23(1):181–92. 10.1111/jch.14129 PMC8029768 33331692

[41] Haze T, Ozawa M, Kawano R, Haruna A, Ohki Y, Suzuki S, et al. Effect of the interaction between the visceral-to-subcutaneous fat ratio and aldosterone on cardiac function in patients with primary aldosteronism. Hypertens Res. 2023;46(5):1132–44. 10.1038/s41440-023-01170-9 36754972

[42] Shen H, Wu Z, Luo W, Chen X, Yang J, Zeng Q, et al. Perirenal Adipose Tissue and Hypertension: Observational and Genetic Analyses. Hypertens (Dallas Tex 1979). 2025;82(11):1809–21. 10.1161/HYPERTENSIONAHA.125.24995 40726397

[43] Mitsuno R, Nakamura T, Nakamura K, Kaneko K, Kojima D, Mizutani Y, et al. Effects of perirenal fat accumulation on cardiometabolic and renal functions and mineralocorticoid receptor activation in primary aldosteronism. Hypertens Res. 2025;48(12):3244–56. 10.1038/s41440-025-02401-x 41057545

[44] Kloock S, Kagan L, Ziegler C, Srivastava M, Tönjes A, Rayes N, et al. Adipose tissue signaling in aldosterone-producing adenomas: paracrine and endocrine effects. Eur J Endocrinol. 2025;193(4):440–52. 10.1093/ejendo/lvaf190 40987749

[45] Costa R, Rodrigues D, Gonçalves T, Alves J, Awata W, Bruder-Nascimento A et al. Autophagy induction protects the perivascular adipose tissue during hyperaldosteronism-induced hypertension. Physiology [Internet]. 2023;38(S1):5734527. Available from: 10.1152/physiol.2023.38.S1.5734527

[46] Costa RM, Bruder-Nascimento A, Alves JV, Awata WMC, Singh S, Rodrigues D et al. Beclin-1-dependent autophagy protects perivascular adipose tissue function from hyperaldosteronism effects. Am J Physiol Circ Physiol. 2025;328(6):H1253–66. 10.1152/ajpheart.00829.2024 40327449

[47] Luther JM. Effects of aldosterone on insulin sensitivity and secretion. Steroids. 2014;91:54–60. 10.1016/j.steroids.2014.08.016 PMC4252580 25194457

[48] Selvaraj J, Sathish S, Mayilvanan C, Balasubramanian K. Excess aldosterone-induced changes in insulin signaling molecules and glucose oxidation in gastrocnemius muscle of adult male rat. Mol Cell Biochem. 2013;372(1–2):113–26. 10.1007/s11010-012-1452-2 23007523

[49] Wada T, Ohshima S, Fujisawa E, Koya D, Tsuneki H, Sasaoka T. Aldosterone inhibits insulin-induced glucose uptake by degradation of insulin receptor substrate (IRS) 1 and IRS2 via a reactive oxygen species-mediated pathway in 3T3-L1 adipocytes. Endocrinology. 2009;150(4):1662–9. 10.1210/en.2008-1018 19095745

[50] Selvaraj J, Muthusamy T, Srinivasan C, Balasubramanian K. Impact of excess aldosterone on glucose homeostasis in adult male rat. Clin Chim Acta. 2009;407(1–2):51–7. 10.1016/j.cca.2009.06.030 19567248

[51] Yamashita R, Kikuchi T, Mori Y, Aoki K, Kaburagi Y, Yasuda K, et al. Aldosterone stimulates gene expression of hepatic gluconeogenic enzymes through the glucocorticoid receptor in a manner independent of the protein kinase B cascade. Endocr J. 2004;51(2):243–51. 10.1507/endocrj.51.243 15118277

[52] Bender SB, McGraw AP, Jaffe IZ, Sowers JR. Mineralocorticoid receptor-mediated vascular insulin resistance: an early contributor to diabetes-related vascular disease? Diabetes. 2013;62(2):313–9. 10.2337/db12-0905 PMC3554383 23349535

[53] Jia G, Lockette W, Sowers JR. Mineralocorticoid receptors in the pathogenesis of insulin resistance and related disorders: from basic studies to clinical disease. Am J Physiol Regul Integr Comp Physiol. 2021;320(3):R276–86. 10.1152/ajpregu.00280.2020 PMC7988773 33438511

[54] Luther JM, Luo P, Kreger MT, Brissova M, Dai C, Whitfield TT, et al. Aldosterone decreases glucose-stimulated insulin secretion in vivo in mice and in murine islets. Diabetologia. 2011;54(8):2152–63. 10.1007/s00125-011-2158-9 PMC3216479 21519965

[55] Giacchetti G, Ronconi V, Turchi F, Agostinelli L, Mantero F, Rilli S, et al. Aldosterone as a key mediator of the cardiometabolic syndrome in primary aldosteronism: an observational study. J Hypertens. 2007;25(1):177–86. 10.1097/HJH.0b013e3280108e6f 17143190

[56] Catena C, Lapenna R, Baroselli S, Nadalini E, Colussi G, Novello M, et al. Insulin sensitivity in patients with primary aldosteronism: a follow-up study. J Clin Endocrinol Metab. 2006;91(9):3457–63. 10.1210/jc.2006-0736 16822818

[57] Kumagai E, Adachi H, Jacobs DRJ, Hirai Y, Enomoto M, Fukami A, et al. Plasma aldosterone levels and development of insulin resistance: prospective study in a general population. Hypertens (Dallas Tex 1979). 2011;58(6):1043–8. 10.1161/HYPERTENSIONAHA.111.180521 22068870

[58] Barrera-Chimal J, Lima-Posada I, Bakris GL, Jaisser F. Mineralocorticoid receptor antagonists in diabetic kidney disease - mechanistic and therapeutic effects. Nat Rev Nephrol. 2022;18(1):56–70. 10.1038/s41581-021-00490-8 34675379

[59] Nedungadi D, Ayodele Adesanya TM, Rayan MN, Zhao S, Williams A, Brock G, et al. The Association of Adiposity and RAAS With Incident Diabetes in African Americans: The Jackson Heart Study. J Clin Endocrinol Metab. 2024;110(1):151–8. 10.1210/clinem/dgae396 PMC11651680 38885313

[60] Hu Y, Zhang J, Liu W, Su X. Determining the Prevalence of Primary Aldosteronism in Patients With New-Onset Type 2 Diabetes and Hypertension. J Clin Endocrinol Metab. 2020;105(4). 10.1210/clinem/dgz293 32163576

[61] Higa M, Ichijo T, Hirose T. Aldosterone-to-Renin Ratio Is Associated with Diabetic Nephropathy in Type 2 Diabetic Patients: A Single-Center Retrospective Study. Med Sci Monit Int Med J Exp Clin Res. 2022;28:e935615. 10.12659/MSM.935615 PMC8944151 35306503

[62] Hannich M, Wallaschofski H, Nauck M, Reincke M, Adolf C, Völzke H, et al. Physiological Aldosterone Concentrations Are Associated with Alterations of Lipid Metabolism: Observations from the General Population. Int J Endocrinol. 2018;2018:4128174. 10.1155/2018/4128174 PMC5892232 29780416

[63] Zhu Q-G, Zhu F. Meta-analysis of blood parameters related to lipid and glucose metabolism between two subtypes of primary aldosteronism. J Clin Hypertens (Greenwich). 2023;25(1):13–21. 10.1111/jch.14607 PMC9832233 36484331

[64] Baudrand R, Gupta N, Garza AE, Vaidya A, Leopold JA, Hopkins PN et al. Caveolin 1 Modulates Aldosterone-Mediated Pathways of Glucose and Lipid Homeostasis. J Am Heart Assoc. 2016;5(10). 10.1161/JAHA.116.003845 PMC5121487 27680666

[65] Otsuka H, Abe M, Kobayashi H. The Effect of Aldosterone on Cardiorenal and Metabolic Systems. Int J Mol Sci. 2023;24(6). 10.3390/ijms24065370 PMC10049192 36982445

[66] Huang BS, Leenen FHH. Mineralocorticoid actions in the brain and hypertension. Curr Hypertens Rep. 2011;13(3):214–20. 10.1007/s11906-011-0192-0 21298576

[67] Ohno Y, Sone M, Inagaki N, Yamasaki T, Ogawa O, Takeda Y, et al. Obesity as a Key Factor Underlying Idiopathic Hyperaldosteronism. J Clin Endocrinol Metab. 2018;103(12):4456–64. 10.1210/jc.2018-00866 30165444

[68] Grewal S, Fosam A, Chalk L, Deven A, Suzuki M, Correa RR, et al. Insulin sensitivity and pancreatic β-cell function in patients with primary aldosteronism. Endocrine. 2021;72(1):96–103. 10.1007/s12020-020-02576-y PMC8087621 33462741

[69] Hernandez-Rubio A, McNally RJ, Pedrós Barnils N, Farukh B, Chowienczyk PJ, Cruickshank JK, et al. Role of Ethnicity and Sex in Hypertension-Mediated Organ Damage in a Dual-Ethnic Cohort of Individuals With Hypertension. Hypertens (Dallas Tex 1979). 2026;83(2):e25032. 10.1161/HYPERTENSIONAHA.125.25032 PMC12822773 41111403

[70] Gonzalez-Sanchez E, Firrincieli D, Housset C, Chignard N. Expression patterns of nuclear receptors in parenchymal and non-parenchymal mouse liver cells and their modulation in cholestasis. Biochim Biophys acta Mol basis Dis. 2017;1863(7):1699–708. 10.1016/j.bbadis.2017.04.004 28390947

[71] Mohib MM, Rabe S, Nolze A, Rooney M, Ain Q, Zipprich A, et al. Eplerenone, a mineralocorticoid receptor inhibitor, reduces cirrhosis associated changes of hepatocyte glucose and lipid metabolism. Cell Commun Signal. 2024;22(1):614. 10.1186/s12964-024-01991-2 PMC11660827 39707386

[72] Queisser N, Happ K, Link S, Jahn D, Zimnol A, Geier A, et al. Aldosterone induces fibrosis, oxidative stress and DNA damage in livers of male rats independent of blood pressure changes. Toxicol Appl Pharmacol. 2014;280(3):399–407. 10.1016/j.taap.2014.08.029 25204689

[73] Habibi J, Chen D, Hulse JL, Whaley-Connell A, Sowers JR, Jia G. Targeting mineralocorticoid receptors in diet-induced hepatic steatosis and insulin resistance. Am J Physiol Regul Integr Comp Physiol. 2022;322(3):R253–62. 10.1152/ajpregu.00316.2021 PMC8896998 35107025

[74] Ramírez-Mejía MM, Qi X, Abenavoli L, Romero-Gómez M, Eslam M, Méndez-Sánchez N. Metabolic dysfunction: The silenced connection with fatty liver disease. Ann Hepatol. 2023;28(6):101138. 10.1016/j.aohep.2023.101138 37468095

[75] Li Z, Luo G, Gan C, Zhang H, Li L, Zhang X et al. Spatially resolved multi-omics of human metabolic dysfunction-associated steatotic liver disease. Nat Genet [Internet]. 2025;57(December). Available from: 10.1038/s41588-025-02407-8 PMC12695644 41286103

[76] Caprio M, Newfell BG, la Sala A, Baur W, Fabbri A, Rosano G, et al. Functional mineralocorticoid receptors in human vascular endothelial cells regulate intercellular adhesion molecule-1 expression and promote leukocyte adhesion. Circ Res. 2008;102(11):1359–67. 10.1161/CIRCRESAHA.108.174235 PMC2597516 18467630

[77] Kirsch T, Beese M, Wyss K, Klinge U, Haller H, Haubitz M et al. Aldosterone modulates endothelial permeability and endothelial nitric oxide synthase activity by rearrangement of the actin cytoskeleton. Hypertens (Dallas, Tex 1979). 2013;61(2):501–8. 10.1161/HYPERTENSIONAHA.111.196832 23213194

[78] Zhang Y-Y, Li C, Yao G-F, Du L-J, Liu Y, Zheng X-J, et al. Deletion of Macrophage Mineralocorticoid Receptor Protects Hepatic Steatosis and Insulin Resistance Through ERα/HGF/Met Pathway. Diabetes. 2017;66(6):1535–47. 10.2337/db16-1354 PMC5860190 28325853

[79] Muñoz-Durango N, Arrese M, Hernández A, Jara E, Kalergis AM, Cabrera D. A Mineralocorticoid Receptor Deficiency in Myeloid Cells Reduces Liver Steatosis by Impairing Activation of CD8(+) T Cells in a Nonalcoholic Steatohepatitis Mouse Model. Front Immunol. 2020;11:563434. 10.3389/fimmu.2020.563434 PMC7772468 33391254

[80] Schreier B, Wolf A, Hammer S, Pohl S, Mildenberger S, Rabe S, et al. The selective mineralocorticoid receptor antagonist eplerenone prevents decompensation of the liver in cirrhosis. Br J Pharmacol. 2018;175(14):2956–67. 10.1111/bph.14341 PMC6016674 29682743

[81] Pizarro M, Solís N, Quintero P, Barrera F, Cabrera D, Rojas-de Santiago P, et al. Beneficial effects of mineralocorticoid receptor blockade in experimental non-alcoholic steatohepatitis. Liver Int Off J Int Assoc Study Liver. 2015;35(9):2129–38. 10.1111/liv.12794 PMC4522413 25646700

[82] Rombouts K, Niki T, Wielant A, Hellemans K, Schuppan D, Kormoss N, et al. Effect of aldosterone on collagen steady state levels in primary and subcultured rat hepatic stellate cells. J Hepatol. 2001;34(2):230–8. 10.1016/s0168-8278(00)00087-8 11281551

[83] Schreier B, Zipprich A, Uhlenhaut H, Gekle M. Mineralocorticoid receptors in non-alcoholic fatty liver disease. Br J Pharmacol. 2022;179(13):3165–77. 10.1111/bph.15784 34935140

[84] Inzaugarat ME, Johnson CD, Holtmann TM, McGeough MD, Trautwein C, Papouchado BG, et al. NLR Family Pyrin Domain-Containing 3 Inflammasome Activation in Hepatic Stellate Cells Induces Liver Fibrosis in Mice. Hepatology. 2019;69(2):845–59. 10.1002/hep.30252 PMC6351190 30180270

[85] Chaturantabut S, Shwartz A, Evason KJ, Cox AG, Labella K, Schepers AG, et al. Estrogen Activation of G-Protein-Coupled Estrogen Receptor 1 Regulates Phosphoinositide 3-Kinase and mTOR Signaling to Promote Liver Growth in Zebrafish and Proliferation of Human Hepatocytes. Gastroenterology. 2019;156(6):1788–e180413. 10.1053/j.gastro.2019.01.010 PMC6532055 30641053

[86] Zucchetti AE, Barosso IR, Boaglio AC, Basiglio CL, Miszczuk G, Larocca MC, et al. G-protein-coupled receptor 30/adenylyl cyclase/protein kinase A pathway is involved in estradiol 17ß-D-glucuronide-induced cholestasis. Hepatology. 2014;59(3):1016–29. 10.1002/hep.26752 24115158

[87] Feng G, Cai J, Huang Y, Zhu X, Gong B, Yang Z, et al. G-Protein-Coupled Estrogen Receptor 1 Promotes Gender Disparities in Hepatocellular Carcinoma via Modulation of SIN1 and mTOR Complex 2 Activity. Mol Cancer Res. 2020;18(12):1863–75. 10.1158/1541-7786.MCR-20-0173 32873626

[88] Fallo F, Dalla Pozza A, Tecchio M, Tona F, Sonino N, Ermani M, et al. Nonalcoholic fatty liver disease in primary aldosteronism: a pilot study. Am J Hypertens. 2010;23(1):2–5. 10.1038/ajh.2009.206 19910932

[89] Tizianel I, Madinelli A, Crimì F, Barbot M, Censi S, Sabbadin C, et al. Prevalence of Metabolic-Associated Steatotic Liver Disease in Patients With Primary Aldosteronism. Clin Endocrinol (Oxf). 2025;102(6):618–25. 10.1111/cen.15231 PMC12046541 40079488

[90] Chen Y, Chen X, Chen Q, Yu C. Non-Alcoholic Fatty Liver Disease and Hypokalemia in Primary Aldosteronism Among Chinese Population. Front Endocrinol (Lausanne). 2021;12:565714. 10.3389/fendo.2021.565714 PMC8101285 33967948

[91] Zeng Q, Luo X, Chen X, Luo W, Li R, Yang S, et al. Renin-independent aldosteronism and metabolic dysfunction-associated steatotic liver disease and cirrhosis: A genetic association study. Clin Nutr. 2025;44:193–200. 10.1016/j.clnu.2024.12.011 39708461

[92] Hu J, Cai X, Zhu Q, Heizhati M, Wen W, Luo Q, et al. Relationship Between Plasma Aldosterone Concentrations and Non-Alcoholic Fatty Liver Disease Diagnosis in Patients with Hypertension: A Retrospective Cohort Study. Diabetes Metab Syndr Obes. 2023;16:1625–36. 10.2147/DMSO.S408722 PMC10257476 37304667

[93] Kumar A, Blackshear C, Subauste JS, Esfandiari NH, Oral EA, Subauste AR. Fatty Liver Disease, Women, and Aldosterone: Finding a Link in the Jackson Heart Study. J Endocr Soc. 2017;1(5):460–9. 10.1210/js.2017-00055 PMC5686785 29264501

[94] Gamliel-Lazarovich A, Raz-Pasteur A, Coleman R, Keidar S. The effects of aldosterone on diet-induced fatty liver formation in male C57BL/6 mice: comparison of adrenalectomy and mineralocorticoid receptor blocker. Eur J Gastroenterol Hepatol. 2013;25(9):1086–92. 10.1097/MEG.0b013e328360554a 23524523

[95] Eshraghian A, Taghavi A, Nikoupour H, Nikeghbalian S, Malek-Hosseini SA. Angiotensin receptor blockers might be protective against hepatic steatosis after liver transplantation. BMC Gastroenterol. 2023;23(1):152. 10.1186/s12876-023-02781-9 PMC10184358 37189076

[96] Ng WH, Yeo YH, Kim H, Seki E, Rees J, Ma KS-K, et al. Renin-angiotensin-aldosterone system inhibitor use improves clinical outcomes in patients with metabolic dysfunction-associated steatotic liver diseases: Target trial emulation using real-world data. Hepatology. 2026;83(2):333–43. 10.1097/HEP.0000000000001333 40178454

[97] Kim KM, Roh J-H, Lee S, Yoon J-H. Clinical implications of renin-angiotensin system inhibitors for development and progression of non-alcoholic fatty liver disease. Sci Rep. 2021;11(1):2884. 10.1038/s41598-021-81959-1 PMC7858633 33536442

[98] Pelusi S, Petta S, Rosso C, Borroni V, Fracanzani AL, Dongiovanni P, et al. Renin-Angiotensin System Inhibitors, Type 2 Diabetes and Fibrosis Progression: An Observational Study in Patients with Nonalcoholic Fatty Liver Disease. PLoS ONE. 2016;11(9):e0163069. 10.1371/journal.pone.0163069 PMC5029872 27649410

[99] Shen D, Song S, Hu J, Cai X, Zhu Q, Zhang Y, et al. The potential of spironolactone to mitigate the risk of nonalcoholic fatty liver disease in hypertensive populations: evidence from a cohort study. Eur J Gastroenterol Hepatol. 2025;37(9):1010–20. 10.1097/MEG.0000000000002986 PMC12316119 40359267

[100] Polyzos SA, Kountouras J, Zafeiriadou E, Patsiaoura K, Katsiki E, Deretzi G, et al. Effect of spironolactone and vitamin E on serum metabolic parameters and insulin resistance in patients with nonalcoholic fatty liver disease. J Renin Angiotensin Aldosterone Syst. 2011;12(4):498–503. 10.1177/1470320311402110 21436212

[101] Perakakis N, Bornstein SR, Birkenfeld AL, Linkermann A, Demir M, Anker SD, et al. Efficacy of finerenone in patients with type 2 diabetes, chronic kidney disease and altered markers of liver steatosis and fibrosis: A FIDELITY subgroup analysis. Diabetes Obes Metab. 2024;26(1):191–200. 10.1111/dom.15305 37814928

[102] Chaudhury CS, Purdy JB, Liu C-Y, Morse CG, Stanley TL, Kleiner D, et al. Unanticipated increases in hepatic steatosis among human immunodeficiency virus patients receiving mineralocorticoid receptor antagonist eplerenone for non-alcoholic fatty liver disease. Liver Int Off J Int Assoc Study Liver. 2018;38(5):797–802. 10.1111/liv.13734 PMC7939026 29509992

[103] O’Shea P, Brady JJ, Gallagher N, Dennedy MC, Fitzgibbon M. Establishment of reference intervals for aldosterone and renin in a Caucasian population using the newly developed Immunodiagnostic Systems specialty immunoassay automated system. Ann Clin Biochem. 2016;53(Pt 3):390–8. 10.1177/0004563215603401 26589630

[104] Toering TJ, Gant CM, van der Visser FW, Laverman GD, Danser AHJ, et al. Sex differences in renin-angiotensin-aldosterone system affect extracellular volume in healthy subjects. Am J Physiol Ren Physiol. 2018;314(5):F873–8. 10.1152/ajprenal.00109.2017 28592435

[105] Szmuilowicz ED, Adler GK, Williams JS, Green DE, Yao TM, Hopkins PN, et al. Relationship between aldosterone and progesterone in the human menstrual cycle. J Clin Endocrinol Metab. 2006;91(10):3981–7. 10.1210/jc.2006-1154 16868049

[106] Baker ME, Katsu Y, Progesterone. An enigmatic ligand for the mineralocorticoid receptor. Biochem Pharmacol. 2020;177:113976. 10.1016/j.bcp.2020.113976 32305433

[107] Rossi GP, Caroccia B, Seccia TM. Role of estrogen receptors in modulating aldosterone biosynthesis and blood pressure. Steroids. 2019;152:108486. 10.1016/j.steroids.2019.108486 31499072

[108] Vasan RS, Evans JC, Benjamin EJ, Levy D, Larson MG, Sundstrom J, et al. Relations of serum aldosterone to cardiac structure: gender-related differences in the Framingham Heart Study. Hypertens (Dallas Tex 1979). 2004;43(5):957–62. 10.1161/01.HYP.0000124251.06056.8e 15007028

[109] Belin de Chantemèle EJ, Mintz JD, Rainey WE, Stepp DW. Impact of leptin-mediated sympatho-activation on cardiovascular function in obese mice. Hypertens (Dallas Tex 1979). 2011;58(2):271–9. 10.1161/HYPERTENSIONAHA.110.168427 PMC3141100 21690486

[110] Hundemer GL, Agharazii M, Madore F, Piché M-E, Gagnon C, Bussières A, et al. Sex-specific Associations of Aldosterone and Renin With Body Composition: A Population-based Cohort Study. J Clin Endocrinol Metab. 2025;110(3):801–10. 10.1210/clinem/dgae566 PMC11834704 39148442

[111] Bansal S, Canziani MEF, Birne R, Anker SD, Bakris GL, Filippatos G, et al. Finerenone cardiovascular and kidney outcomes by age and sex: FIDELITY post hoc analysis of two phase 3, multicentre, double-blind trials. BMJ Open. 2024;14(3):e076444. 10.1136/bmjopen-2023-076444 PMC10952937 38508632

[112] Morán-Costoya A, Proenza AM, Gianotti M, Lladó I, Valle A. Sex Differences in Nonalcoholic Fatty Liver Disease: Estrogen Influence on the Liver-Adipose Tissue Crosstalk. Antioxid Redox Signal. 2021;35(9):753–74. 10.1089/ars.2021.0044 33736456

[113] Stokar J, Dresner-Pollak R. Earlier Menopause and Risk of Metabolic Dysfunction-Associated Steatotic Liver Disease: A Global Cohort Study. Diabetes Metab Res Rev. 2026;42(1):e70121. 10.1002/dmrr.70121 PMC12790266 41518227

[114] Kim D, Manikat R, Cholankeril G, Ahmed A. Endogenous sex hormones and nonalcoholic fatty liver disease in US adults. Liver Int Off J Int Assoc Study Liver. 2024;44(2):460–71. 10.1111/liv.15786 38010926

[115] Agarwal R, Filippatos G, Pitt B, Anker SD, Rossing P, Joseph A, et al. Cardiovascular and kidney outcomes with finerenone in patients with type 2 diabetes and chronic kidney disease: the FIDELITY pooled analysis. Eur Heart J. 2022;43(6):474–84. 10.1093/eurheartj/ehab777 PMC8830527 35023547

[116] Ito S, Itoh H, Rakugi H, Okuda Y, Yoshimura M, Yamakawa S. Double-Blind Randomized Phase 3 Study Comparing Esaxerenone (CS-3150) and Eplerenone in Patients With Essential Hypertension (ESAX-HTN Study). Hypertens (Dallas Tex 1979). 2020;75(1):51–8. 10.1161/HYPERTENSIONAHA.119.13569 31786983

[117] Bakris G, Yang YF, Pitt B. Mineralocorticoid Receptor Antagonists for Hypertension Management in Advanced Chronic Kidney Disease: BLOCK-CKD Trial. Hypertens (Dallas Tex 1979). 2020;76(1):144–9. 10.1161/HYPERTENSIONAHA.120.15199 32520623

[118] Freeman MW, Halvorsen Y-D, Marshall W, Pater M, Isaacsohn J, Pearce C, et al. Phase 2 Trial of Baxdrostat for Treatment-Resistant Hypertension. N Engl J Med. 2023;388(5):395–405. 10.1056/NEJMoa2213169 36342143

[119] Saxena M, Laffin L, Borghi C, Fernandez Fernandez B, Ghali JK, Kopjar B, et al. Lorundrostat in Participants With Uncontrolled Hypertension and Treatment-Resistant Hypertension: The Launch-HTN Randomized Clinical Trial. JAMA. 2025;334(5):409–18. 10.1001/jama.2025.9413 PMC12210145 40587141

[120] Mulatero P, Wuerzner G, Groessl M, Sconfienza E, Damianaki A, Forestiero V, et al. Safety and efficacy of once-daily dexfadrostat phosphate in patients with primary aldosteronism: a randomised, parallel group, multicentre, phase 2 trial. EClinicalMedicine. 2024;71:102576. 10.1016/j.eclinm.2024.102576 PMC11015343 38618204

[121] Schulze F, Schaible J, Goettel M, Tanaka Y, Hohl K, Schultz A, et al. Phase 1 studies of the safety, tolerability, pharmacokinetics, and pharmacodynamics of BI 690517 (vicadrostat), a novel aldosterone synthase inhibitor, in healthy male volunteers. Naunyn Schmiedebergs Arch Pharmacol. 2025;398(7):9083–98. 10.1007/s00210-025-03838-0 PMC12263707 39899058

